# Effects of the acid–base treatment of corn on rumen fermentation and microbiota, inflammatory response and growth performance in beef cattle fed high-concentrate diet

**DOI:** 10.1017/S1751731120000786

**Published:** 2020-09

**Authors:** J. Liu, K. Tian, Y. Sun, Y. Wu, J. Chen, R. Zhang, T. He, G. Dong

**Affiliations:** Department of Animal Science, College of Animal Science and Technology, Southwest University, Beibei, Chongqing 400715, P. R. China

**Keywords:** hydrochloric acid, sodium bicarbonate, ruminal bacteria, lipopolysaccharide, steer

## Abstract

Beef cattle are often fed high-concentrate diet (**HCD**) to achieve high growth rate. However, HCD feeding is strongly associated with metabolic disorders. Mild acid treatment of grains in HCD with 1% hydrochloric acid (**HA**) followed by neutralization with sodium bicarbonate (**SB**) might modify rumen fermentation patterns and microbiota, thereby decreasing the negative effects of HCD. This study was thus aimed to investigate the effects of treatment of corn with 1% HA and subsequent neutralization with SB on rumen fermentation and microbiota, inflammatory response and growth performance in beef cattle fed HCD. Eighteen beef cattle were randomly allocated to three groups and each group was fed different diets: low-concentrate diet (**LCD**) (concentrate : forage = 40 : 60), HCD (concentrate : forage = 60 : 40) or HCD based on treated corn (**HCDT**) with the same concentrate to forage ratio as the HCD. The corn in the HCDT was steeped in 1% HA (wt/wt) for 48 h and neutralized with SB after HA treatment. The animal trial lasted for 42 days with an adaptation period of 7 days. At the end of the trial, rumen fluid samples were collected for measuring ruminal pH values, short-chain fatty acids, endotoxin (or lipopolysaccharide, **LPS**) and bacterial microbiota. Plasma samples were collected at the end of the trial to determine the concentrations of plasma LPS, proinflammatory cytokines and acute phase proteins (**APPs**). The results showed that compared with the LCD, feeding the HCD had better growth performance due to a shift in the ruminal fermentation pattern from acetate towards propionate, butyrate and valerate. However, the HCD decreased ruminal pH and increased ruminal LPS release and the concentrations of plasma proinflammatory cytokines and APPs. Furthermore, feeding the HCD reduced bacterial richness and diversity in the rumen. Treatment of corn increased resistant starch (**RS**) content. Compared with the HCD, feeding the HCDT reduced ruminal LPS and improved ruminal bacterial microbiota, resulting in decreased inflammation and improved growth performance. In conclusion, although the HCD had better growth performance than the LCD, feeding the HCD promoted the pH reduction and the LPS release in the rumen, disturbed the ruminal bacterial stability and increased inflammatory response. Treatment of corn with HA in combination with subsequent SB neutralization increased the RS content and helped counter the negative effects of feeding HCD to beef steers.

## Implications

Beef cattle are oftentimes fed high-concentrate diet in the fattening stage to provide more energy for achieving higher growth rate. However, feeding high-concentrate diet often leads to lower rumen fluid pH, increased release of endotoxin and microbiota dysbiosis in the rumen. Treatment of corn in the high-concentrate diet with 1% hydrochloric acid in combination with subsequent sodium bicarbonate neutralization modified rumen fermentation patterns and microbiota and helped counter the negative effects of feeding a high-concentrate diet to beef steers. The results of the study provide an insight into the avenues for controlling high-concentrate-diet-associated problems in beef cattle production.

## Introduction

In practical beef production, cattle are often fed high-concentrate diet (**HCD**) to meet high energy demand in order to achieve maximum production (Ye *etal*., 2016). High-concentrate diet is rich in carbohydrate that can lead to rapid ruminal production of organic acids like short-chain fatty acids (**SCFAs**) which are the major energy source for the ruminant host (McCann *et al.,* 2016). The rapid accumulation of SCFAs reduced buffering capacity in the rumen and could result in subacute ruminal acidosis (**SARA**) (McCann *et al.*, 2016). In the meantime, the bacterial community structure and diversity in the rumen were altered (Mao *et al.*, 2016). Consequently, lipopolysaccharide (**LPS**), a cell wall component of gram-negative bacteria, was released in an increasing amount (Eckel and Ametaj, 2016; Plaizier *et al.*, 2017). Lipopolysaccharide can translocate into the circulation across the epithelium of the digestive tract to cause inflammation response (Khafipour *etal*., 2009a; Dong *etal*., 2011).

In order to alleviate the decrease of rumen pH caused by rapid degradation of large amount of starch in HCD, many researchers have tried to treat grains to reduce starch degradation in the rumen using chemical methods to increase starch resistance against being digested by rumen microbial glycosidases (Deckardt *et al.*, 2013). Of these methods, lactic acid has been used to treat grains to promote resistant starch (**RS**) formation and to reduce the rate of starch digestion in the rumen (Liljeberg *et al.,* 1995; Hallstrom *et al.*, 2011).Metzler-Zebeli *et al.* (2018) reported that steeping by-product concentrate of a by-product-rich diet in 5% lactic acid considerably changed the rumen bacterial community composition and increased the bacterial richness and diversity. In the study of Iqbal *et al.* (2010), steeping barely grain in 0.5% lactic acid decreased the rate of starch degradation in the rumen and minimized the risk of SARA through modulating ruminal fermentation. They also demonstrated that the content of soluble starch of barely grain steeped in 0.5% lactic acid decreased and RS increased by 17.7% (Iqbal *etal*., 2009). According to a previous study, increasing RS content to reduce fermentation and avoid the accumulation of SCFA in rumen is an effective method to reduce the risk of SARA (Deckardt *et al.*, 2013).

Hydrochloric acid (**HA**) is an important component of the gastric juice secreted by the abomasum of ruminant animals. In our previous research, we found that treating corn in HCD with 1% HA for 48 h improved ruminal degradation characteristics and decreased inflammatory response in beef cattle (Yang *et al.,* 2018a). However, the mode of action of the HA-treated corn is unclear. We hypothesized that the treatment of corn with 1% HA will modulate rumen fermentation and improve ruminal bacterial microbiota through increasing RS when a HCD is fed, and this will minimize ruminal LPS release and thus reduce systemic inflammatory response. Furthermore, we assume that the neutralization with sodium bicarbonate (**SB**) after the HA treatment will eliminate the negative effect of acidity on ruminal pH. Thus, the objective of this study was to investigate the effects of treatment of corn with 1% HA for 48 h and with subsequent neutralization by SB on ruminal fermentation and microbiota, inflammatory response and growth performance in finishing beef steers fed HCD.

## Material and methods

### Animals, diets and management

Eighteen Simmental × Luxi hybrid steers with an initial BW of 318 ± 9.4 kg were selected for this experiment. The cattle were fed the same diet and nursed in the same condition before the start of the trial. Rumen fluid samples were collected from all animals to determine the rumen bacterial composition to ensure the baseline bacterial microbiota was identical (Supplementary Tables S1 and S2). Subsequently, the cattle were randomly assigned to three groups (*n* = 6/group) and fed different diets: low-concentrate diet (**LCD**; concentrate : forage = 40 : 60), HCD (concentrate : forage = 60 : 40) or HCD based on treated corn (**HCDT**; concentrate : forage = 60 : 40). A hammer mill with round screen holes of 2.5 mm was used to grind the corn (a yellow variety, CP 8.0%). The ground corn in the HCD and LCD was steeped in tap water in a 1 : 1 ratio (wt/wt) for 48 h. The ground corn of the HCDT was steeped in 1% HA in a 1 : 1 ratio (wt/wt) for 48 h and neutralized with SB in a ratio of 1 : 2.7 (wt/wt) after the HA treatment to achieve a pH of 7. The 1% HA provided by chemical manufacturers is highly diluted acid, which is essentially similar to the HA-containing gastric juice. The treated corn was completely neutralized with SB to eliminate any negative effect of acidity on ruminal pH. The wet corn without removal of the aqueous part after the treatment was included into the total mixed ration. The acid–base treatment used in the study did not pose a threat to the health of animals and the environment. The ingredients and nutrient composition of experimental diets are shown in Table 1. The animal experiment lasted for 42 days with an adaption period of 7 days. The cattle were housed in individual tie stalls and offered diet twice daily at 0700 and 1700 h, with free access to diet and water. After the experiment, all cattle were kept by stockpersons of the university experimental station.

**Table 1:** Ingredients and nutrient composition of the experiment diets fed to beef cattle

	Diets		
	LCD^1^	HCD	HCDT
Ingredients (g/kg, DM)			
Rice straw	305.0	105.0	105.0
Sorghum distiller’s grains	295.0	295.0	295.0
Corn grain, untreated	297.6	497.6	–
Corn grain, treated	–	–	497.6
Rapeseed meal	86.1	86.1	86.1
Salt	3.0	3.0	3.0
Limestone meal	10.5	10.5	10.5
Premix^2^	2.8	2.8	2.8
Nutrient composition (g/kg, DM)^3^			
Metabolizable energy (MJ/kg)	9.62	10.25	10.25
Net energy for maintenance (MJ/kg)	6.51	6.90	6.90
Net energy for growth (MJ/kg)	3.85	4.38	4.38
CP	126	134	134
EE	51	55	55
CF	132	90	90
NDF	438	364	364
ADF	246	161	161
Ca	5.4	5.3	5.3
TP	3.5	3.8	3.8

EE = ether extract; CF = crude fiber; TP = total phosphorus.

1 LCD, low-concentrate diet based on corn steeped in tap water for 48 h; HCD, high-concentrate diet based on corn steeped in tap water for 48 h; HCDT, high-concentrate diet based on corn steeped in 1% (wt/wt) hydrochloric acid for 48 h in combination with subsequent sodium bicarbonate neutralization.

2 Premix provide (per kg): Fe (as ferrous sulfate) 50 mg, Cu (as copper sulfate) 10 mg, Zn (as zinc sulfate) 30 mg, Mn (as sulfate) 20 mg, Co (as chloride) 0.1 mg, I (as iodate) 0.5 mg, Se (as selenite) 0.1 mg, vitamin A 2240 IU, vitamin D_3_ 280 IU and vitamin E 30 IU.

3 Energy contents were calculated based on the China Feed Databank (Chinese Academy of Agricultural Sciences Institute of Animal Science and Chinese Animal Nutrition Association, 1985).

### Sample collection

Corn samples were collected after steeping in either tap water for 48 h for the LCD and HCD or 1% HA for 48 h followed by SB neutralization for the HCDT to measure RS and total starch contents.

Blood samples (15 ml/sample) were taken from the jugular vein before the morning feeding on day 35. Blood sampling was conducted in a gentle and quick manner to minimize pain of the animals. Samples were collected into pyrogen-free tubes containing sodium heparin and centrifuged at 3000×**g** and 4°C for 15 min to harvest plasma, which was stored at −20°C for further analysis of LPS, acute phase proteins (**APPs**) and cytokines.

Rumen fluid samples were collected at the beginning and end of the experiment by an oral stomach tube equipped with a pyrogen-free syringe at 3 h after the morning feeding, as described by Zhou *et al.* (2014). To avoid contamination by saliva, the initial 50-ml rumen fluid was discarded. Subsequently, 150-ml rumen fluid was collected and divided into two portions. One portion was transferred into 15-ml sterile tubes and immediately stored in liquid N for analysis of bacterial composition. Another portion was filtered through four layers of sterile cheesecloth and transferred into depyrogenated centrifuge tubes (50 ml) for LPS and SCFA detection. The pH value of the filtrate was immediately measured using a portable pH meter (Rex PHS-3 E; Shanghai INESA Scientific Instrument Co., Ltd, Shanghai, China).

### Total starch and resistant starch

The RS and total starch were determined using the RS Assay Kit (Megazyme International Ireland Ltd, Wicklow, Ireland) according to the methods of Association of Official Analytical Chemists (2002) and American Association of Cereal Chemists (2010; Method 32–40).Briefly, 0.5-g wet sample was put into a glass tube with a screw cap. And 4-ml pancreatic α-amylase solution containing amyloglucosidase (3 units/ml) was added into each tube. The mixture was incubated at 37°C with continued shaking for 16 h. Then 4-ml ethanol (99% v/v) was added and centrifuged at 1500×**g** for 10 min. The supernatant was collected and mixed with 2-ml ethanol (50%) and stirred up by a vortex mixer. After 6-ml ethanol (50%) was added, it was then centrifuged at 1500×**g** for 10 min. The supernatant was non-resistant starch (**NRS**) and the sediment was RS. The supernatant and the dissolved sediment in KOH were incubated after adding amyloglucosidase and glucose oxidase according to the manufacturer’s protocols. The solution absorbance at 510 nm was measured, and the contents of NRS, RS and total starch were calculated following the manufacturer’s instructions.

### Growth performance

The initial and final BWs were measured for two consecutive days and daily feed intake was recorded. The data were used to calculate average daily gain (**ADG**), average daily DM intake (**ADMI**) and feed : gain ratio (**ADMI/ADG**).

### Short-chain fatty acids

The filtrated rumen fluid was centrifuged at 10 000×**g** and 4°C for 45 min, and the supernatant was collected to measure SCFAs, including acetate, propionate, butyrate, iso-butyrate, valerate and iso-valerate. The supernatant and metaphosphoric acid (25%) were mixed in a ratio of 5 : 1 and placed at 5°C for 5 h. The mixture was centrifuged at 14 500×**g** and 4°C for 15 min, and the supernatant was subjected to gas chromatograph analysis. The procedures of gas chromatograph were the same as those of our previous study (Yang *et al.*, 2018a).

### Lipopolysaccharides, cytokines and acute phase proteins

The LPS concentrations of rumen fluid and plasma were detected using the End-point Chromogenic Tachypleus Amebocyte Lysate kit (Xiamen Bioendo Technology Co., Ltd, Xiamen, China) according to manufacturer’s instructions. The plasma was diluted 10-fold to attain an LPS concentration range of 0.01 to 0.1 endotoxin units (EU)/ml (1 EU = 0.1 ng) and the diluted plasma was incubated at 70°C for 10 min, then cooled in ice water for 3 min before the LPS assay. The rumen fluid was thawed and centrifuged at 10 000×**g** for 15 min, then the supernatant was transferred into another depyrogenated centrifuge tube and recentrifuged at 10 000×**g** for 30 min. The supernatant was filtered through a 0.22-μm pyrogen-free filter (Millex; Millipore Corporation, Bedford, MA, USA) and collected into a depyrogenated glass tube. It was then heated at 100°C for 30 min, cooled at room temperature (about 25°C) for 10 min and subsequently diluted using pyrogen-free water to attain an LPS concentration range of 0.1 to 1 EU/ml, as suggested by the manufacturer before the assay.

Plasma cytokines including interleukin (**IL**)-1β, IL-6, IL-8 and tumor necrosis factor-alpha (**TNF-α**) were determined using the bovine ELISA kit (Sinobest Biotechnology Co., Ltd, Shanghai, China). The APPs including C-reactive protein (**CRP**), LPS-binding protein (**LBP**), haptoglobin (**Hp**) and serum amyloid A (**SAA**) were assayed using the bovine ELISA kit (Shanghai Jinma Biotechnology Co., Ltd, Shanghai, China). The protocols were described in our previous study (Tian *etal*., 2019).

### DNA extraction and PCR amplification

The methodology used for DNA extraction of rumen fluid samples and the measurements of the quantity and quality of extracted DNAs was described in detail in our previous study (Tian *etal*., 2019). Polymerase chain reaction amplification of the bacterial 16S rRNA genes’ V3 to V4 regions was performed using the forward primer 338F (5′-ACTCCTACGGGAGGCAGCA-3′) and the reverse primer 806R (5′-GGACTACHVGGGTWTCTAAT-3′). The PCR amplification was performed as described in our previous study (Tian *etal*., 2019).

### Library construction, sequencing and sequence data processing

The DNA fragments were end-repaired to construct DNA library. Before the sequencing, the successfully constructed DNA library was checked for quality and quantified. The sequenced raw data were quality-filtered by the Sliding Window method. The detailed protocols of library construction, sequencing and sequence data processing were as described in our previous study (Tian *etal*., 2019). The raw sequencing data were submitted to the Sequence Read Archive of National Center for Biotechnology Information (http://www.ncbi.nlm.nih.gov/sra) under the accession number of PRJNA545031.

### Operational taxonomic unit classification

Quantitative Insights Into Microbial Ecology and UCLUST were combined to cluster the high-quality sequences into operational taxonomic units (**OTUs**) at 97% similarity (Caporaso *etal*, 2010; Edgar, 2010). The most abundant sequence in each OTU was selected as the representative sequence. Subsequently, according to the number of sequences in each OTU and each sample, the OTU tables were constructed. Community diversity and richness were estimated utilizing the ACE, Chao1, Simpson and Shannon indices. Principal coordinate analysis (**PCoA**) was conducted by unweighted UniFrac distance method to observe the difference between groups. The detailed procedures were described in our previous study (Tian *etal*., 2019).

### Statistical analyses

Data of the present study were analyzed by one-way ANOVA using SPSS software (version 23) according to the following model: *Y*_*ij*_ = *μ* + *D*_*i*_ + *C*_*j*_ + *e*_*ij*_, where *Y*_*ij*_ is the observation of dependent variables; *μ* is the overall mean; *D*_i_ represents the fixed effect of treatment; *C*_*j*_ is the random cattle effect and *e*_*ij*_ is the residual error for the observation. Differences among treatment means were classified using Duncan’s multiple range test. *P* < 0.05 was considered statistically significant, whereas a tendency was considered at 0.05 *< P* < 0.10.

## Results

### Resistant starch contents

**Figure 1:**
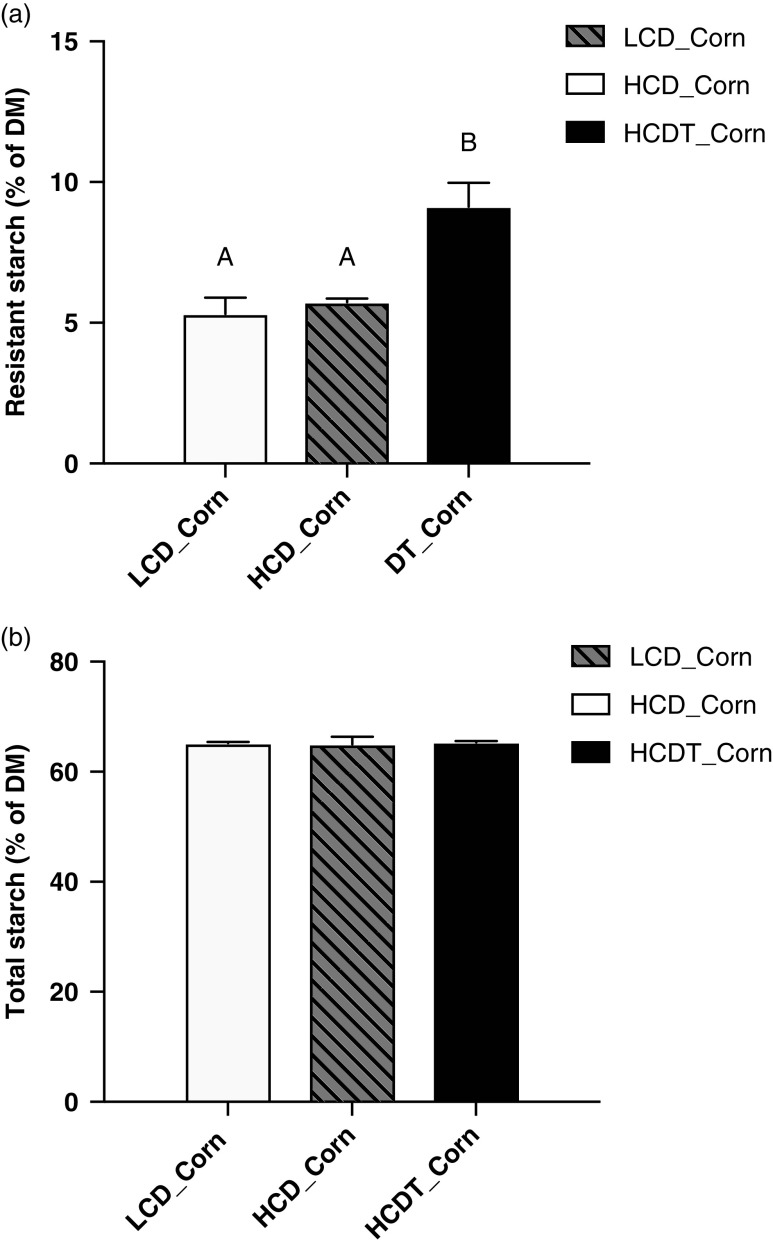
The content of resistant starch (a) and total starch (b) of corn included in different diets fed to beef cattle. LCD_Corn represents corn included in the low-concentrate diet (LCD) and steeped in tap water for 48 h; HCD_Corn represents corn included in the high-concentrate diet (HCD) and steeped in tap water for 48 h; HCDT_Corn represents corn included in the HCD and steeped in 1% (wt/wt) hydrochloric acid for 48 h in combination with subsequent sodium bicarbonate neutralization. Data are presented as the mean ± SD. A,B: Means without a common letter differ (*P* < 0.01).

As shown in Figure 1a, the RS content of the corn after treatment with 1% HA was 9.12%, which was 65% higher (*P* < 0.05) than that of the corn steeped in tap water. Total starch content in the corn was not affected (*P* > 0.05) by the treatment (Figure 1b).

### Ruminal pH and short-chain fatty acids

The rumen fluid pH and SCFA concentrations are shown in Table 2. Feeding HCDs, whether the corn is treated (in the HCDT) or not (in the HCD), led to lower (*P* < 0.01) pH values. Feeding high-concentrate diets (HCD and HCDT) shifted the fermentation pattern from acetate towards propionate, butyrate and valerate. Compared with the LCD and HCD, the HCDT reduced numerically the total SCFA in the rumen fluid, although the difference did not attain a significant level.

**Table 2:** Ruminal pH, short-chain fatty acid (SCFA) concentrations in rumen fluid and lipopolysaccharide (LPS) concentrations in rumen fluid and plasma in beef cattle after feeding different diets

Item	Diets^1^			SEM	*P-*values
	LCD	HCD	HCDT		
Ruminal pH	7.29^A^	6.77^B^	6.90^B^	0.058	<0.001
SCFA concentration (mmol/l)					
Total SCFA	52.54	54.84	51.89	0.646	0.146
Acetate	36.22^A^	26.72^B^	27.03^B^	1.189	<0.001
Propionate	9.59^A^	17.80^B^	15.83^B^	0.934	<0.001
Butyrate	5.51^A^	8.33^B^	7.31^B^	0.339	<0.001
Iso-butyrate	0.21^a^	0.17^b^	0.20^ab^	0.008	0.039
Valerate	0.49^A^	1.07^B^	0.96^B^	0.068	<0.001
Iso-valerate	0.53^a^	0.76^b^	0.56^a^	0.043	0.039
LPS concentration					
Ruminal LPS (EU/ml)^2^	23881^A^	87978^B^	72092^C^	7290.2	<0.001
Plasma LPS (EU/ml)	0.07^A^	0.20^B^	0.18^B^	0.140	<0.001

1 LCD, low-concentrate diet based on corn steeped in tap water for 48 h; HCD, high-concentrate diet based on corn steeped in tap water for 48 h; HCDT, high-concentrate diet based on corn steeped in 1% (wt/wt) hydrochloric acid for 48 h in combination with subsequent sodium bicarbonate neutralization.

2 Endotoxin unit (1 EU = 0.1 ng).

^A,B^Means of the same row not sharing an uppercase letter differ (*P* < 0.01).

^a,b^Means of the same row not sharing a lowercase letter differ (*P* < 0.05).

### Lipopolysaccharide concentration in rumen fluid and plasma

The HCD group had higher LPS content in rumen fluid (*P* < 0.01) and plasma (*P* < 0.01) than the LCD group (Table 2). Treatment of the corn in the HCDT group resulted in a lower ruminal LPS, as compared with the HCD group. The plasma LPS of the HCDT group was numerically lower than that of the HCD group (*P* = 0.071) although the difference was not statistically significant.

### The cytokines and acute phase protein concentrations in plasma

The concentrations of APPs were shown in Table 3. Feeding the HCD significantly increased the LBP, SAA, CRP and Hp concentrations (*P* < 0.05) compared with feeding the LCD. Treatment of the corn in the HCDT resulted in lower concentrations of SAA (*P* < 0.01) and Hp (*P* < 0.05) compared with the HCD group. Plasma cytokines (TNF-α, IL-1β, IL-6 and IL-8) of the HCD group were higher (*P* < 0.05) compared with the LCD group (Table 3). Treatment of the corn in the HCDT group decreased (*P* < 0.05) the TNF-α and IL-6 levels compared with the HCD group.

**Table 3:** The plasma acute phase protein (APP) and proinflammatory cytokine levels in beef cattle fed different diets

Item	Diets^1^			SEM	*P*-values
	LCD	HCD	HCDT		
APP					
LBP (μg/ml)	298^a^	324^b^	309^ab^	5.1	0.013
SAA (μg/ml)	13.7^A^	15.3^B^	13.9^A^	0.24	0.006
CRP (mg/l)	9.4^a^	11.0^b^	9.8^ab^	0.26	0.035
Hp (μg/ml)	1809^a^	2119^b^	1798^a^	60.0	0.034
Proinflammatory cytokine					
TNF-α (pg/ml)	1209^a^	1430^Bb^	1233^Aa^	34.8	0.008
IL-1β (pg/ml)	134^a^	159^b^	157^b^	4.3	0.026
IL-6 (pg/ml)	298^a^	3212^b^	298^a^	4.5	0.038
IL-8 (pg/ml)	291^a^	325^b^	306^ab^	5.9	0.046

LBP = lipopolysaccharide-binding protein; SAA = serum amyloid A; CRP = C-reactive protein; Hp = haptoglobin; TNF-α = tumor necrosis factor α; IL-1β = interleukin-1β; IL-6 = interleukin-6; IL-8 = interleukin-8.

1 LCD, low-concentrate diet based on corn steeped in tap water for 48 h; HCD, high-concentrate diet based on corn steeped in tap water for 48 h; HCDT, high-concentrate diet based on corn steeped in 1% (wt/wt) hydrochloric acid for 48 h in combination with subsequent sodium bicarbonate neutralization.

^A,B^Means of the same row not sharing an uppercase letter differ (*P* < 0.01).

^a,b^Means of the same row not sharing a lowercase letter differ (*P* < 0.05).

### Rumen fluid bacterial community diversity and composition

In total, 587 609 high-quality sequences were generated from 18 rumen fluid samples with an average of 32 645 ± 3759 sequences per sample. Sequences were clustered into OTUs according to the sequence similarity level of 97%. Data of OTU numbers and alpha diversity indices are shown in Table 4. Compared with the LCD, the HCD reduced OTU numbers and all alpha indices (*P* < 0.05). However, feeding the HCDT increased (*P* < 0.05) OTU numbers and almost all alpha indices except the Simpson index, as compared with feeding the HCD.

**Table 4:** Operational taxonomic unit (OTU) and alpha diversity indices of rumen fluid bacterial community in beef cattle fed different diets

Item	Diets^1^			SEM	*P-*values
	LCD	HCD	HCDT		
OTU	1210^a^	1073^Ab^	1258^Ba^	27.0	0.005
Chao1	1446^A^	1261^B^	1578^A^	39.7	0.001
ACE	1448^a^	1289^Ab^	1632^Bc^	42.2	<0.001
Simpson	0.99^A^	0.97^B^	0.98^AB^	0.004	0.026
Shannon	8.38^A^	7.57^B^	8.24^A^	0.116	0.001

1 LCD, low-concentrate diet based on corn steeped in tap water for 48 h; HCD, high-concentrate diet based on corn steeped in tap water for 48 h; HCDT, high-concentrate diet based on corn steeped in 1% (wt/wt) hydrochloric acid for 48 h in combination with subsequent sodium bicarbonate neutralization.

^A,B,C^Means of the same row not sharing an uppercase letter differ (*P* < 0.01).

^a,b,c^Means of the same row not sharing a lowercase letter differ (*P* < 0.05).

Results of the PCoA based on unweighted UniFrac distances illustrated the variation of rumen fluid microbial communities across the diets (Supplementary Figure S1). The PCoA axis 1 (PC1) accounted for 21.35% of the variation and the axis 2 (PC2) accounted for 9.83% of the variation. There was a clear boundary between the LCD and HCD clusters, revealing that the bacterial structures were different between these two groups. The HCDT cluster was not completely separated from that of the LCD or HCD and had some overlaps especially with the HCD group.

**Figure 2:**
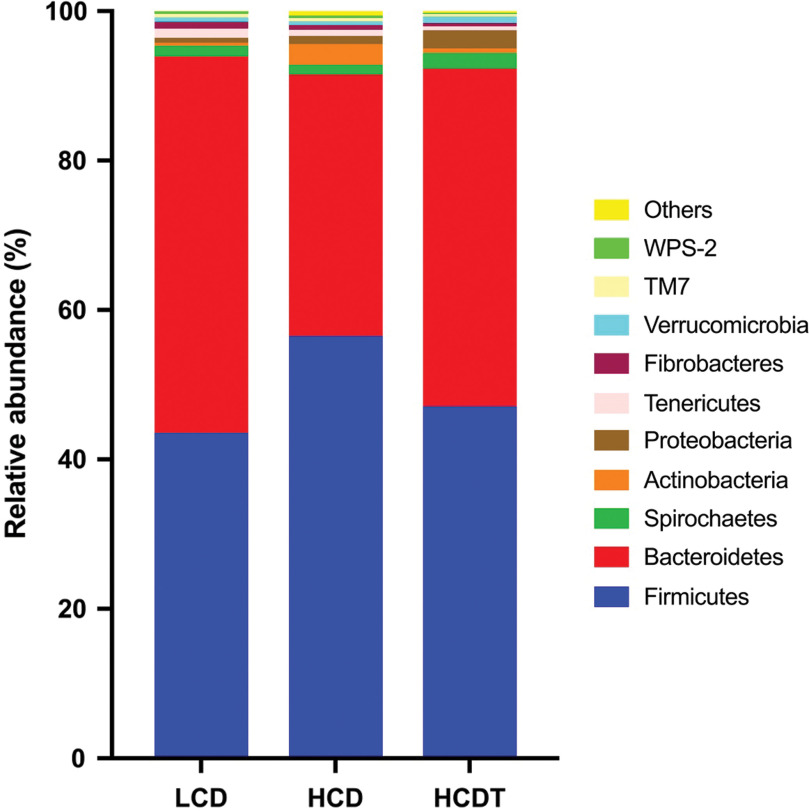
Bacterial composition in rumen fluid at the phylum level when beef cattle were fed different diets. Only the top 10 abundant phyla are presented, and other phyla were pooled into ‘Others’. LCD, low-concentrate diet based on corn steeped in tap water for 48 h; HCD, high-concentrate diet based on corn steeped in tap water for 48 h; HCDT, high-concentrate diet based on corn steeped in 1% (wt/wt) hydrochloric acid for 48 h in combination with subsequent sodium bicarbonate neutralization.

Across the three groups, the two most abundant bacterial phyla in the rumen fluid were Firmicutes and Bacteroidetes (Figure 2; Supplementary Table S3). The third most abundant bacterial phyla of the LCD, HCD and HCDT groups were Spirochaetes, Actinobacteria and Proteobacteria, respectively. The LCD had higher (*P* < 0.01) Bacteroidetes and lower (*P* < 0.01) Firmicutes compared with the HCD and HCDT. The HCDT increased (*P* < 0.01) the abundance of Bacteroidetes compared with the HCD. Moreover, the HCD group had the most abundant Actinobacteria among the three groups (*P* < 0.05).

**Figure 3:**
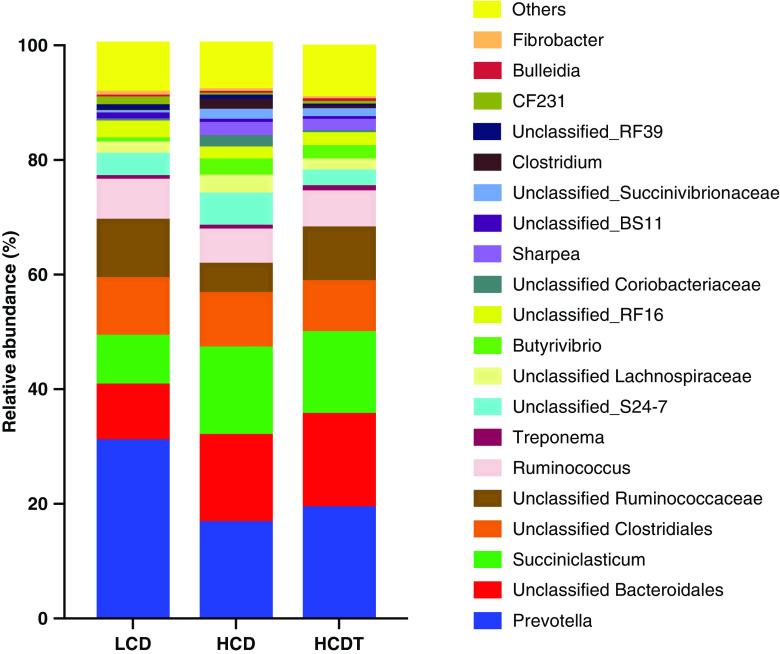
Bacterial composition in rumen fluid at the genus level when beef cattle were fed different diets. Only the top 20 abundant genera are presented, and other genera were pooled into ‘Others’. LCD, low-concentrate diet based on corn steeped in tap water for 48 h; HCD, high-concentrate diet based on corn steeped in tap water for 48 h; HCDT, high-concentrate diet based on corn steeped in 1% (wt/wt) hydrochloric acid for 48 h in combination with subsequent sodium bicarbonate neutralization.

As shown in Figure 3 and Supplementary Table S4, at the genus level, *Prevotella* and *Succiniclasticum* were dominant genera in the rumen fluid across all the groups. Compared with the LCD, the HCD and HCDT decreased (*P* < 0.05) the abundance of *Prevotella* but increased that of *Succiniclasticum* (*P* < 0.05). The percentages of these two genera were not different between the HCD and HCDT groups (*P* > 0.05). Relative abundances of *Butyrivibrio* were significantly increased (*P* < 0.05) in HCDs, whether corn was treated or not, compared with the LCD. Feeding the HCDT increased (*P* < 0.01) Unclassified Ruminococcaceae abundance and decreased (*P* < 0.05) that of Unclassified S24-7 and Unclassified Lachnospiraceae compared with feeding the HCD.

### Growth performance

Final BW, ADG, ADMI and the feed : gain ratio in the HCD and HCDT groups were significantly higher (*P* < 0.05) than in the LCD group (Table 5). Treatment of corn increased final BW and the feed : gain ratio and tented to increase ADG in the HCDT group compared with the HCD group.

**Table 5:** Effects of feeding different diets on growth performance of beef cattle

Item	Diets^1^			SEM	*P*-values
	LCD	HCD	HCDT		
Initial BW (kg)	314	320	319	2.3	0.607
Final BW (kg)	329^Aa^	342^b^	345^Bb^	2.5	0.016
ADG (kg)	0.36^A^	0.52^B^	0.63^B^	0.034	<0.001
ADMI (kg)	6.43^Aa^	6.71^b^	6.96^Bb^	0.071	0.003
Feed : gain	18.34^Aa^	13.76^b^	11.12^Bb^	0.981	0.003

ADG = average daily gain; ADMI = average daily DM intake.

1 LCD, low-concentrate diet based on corn steeped in tap water for 48 h; HCD, high-concentrate diet based on corn steeped in tap water for 48 h; HCDT, high-concentrate diet based on corn steeped in 1% (wt/wt) hydrochloric acid for 48 h in combination with subsequent sodium bicarbonate neutralization.

^A,B^Means of the same row not sharing an uppercase letter differ (*P* < 0.01).

^a,b^Means of the same row not sharing a lowercase letter differ (*P* < 0.05).

## Discussion

In the present study, feeding the HCD significantly increased propionate and butyrate production in the rumen compared with feeding the LCD. It is generally known that propionate and butyrate are more efficiently used for growth than acetate in ruminant fattening (McDonald *etal*., 2011). Therefore, a shift in the rumen fermentation pattern resulting from feeding the HCD contributed to higher growth performance observed in the present study.

However, our results showed that feeding the HCD led to lower pH values and higher LPS release in the rumen. This is consistent with the results of our previous studies and other studies (Mao *etal*., 2013; Yang *et al.*, 2018b). Lipopolysaccharide is the component of the cell wall of gram-negative bacteria, which is increasingly released when gram-negative bacteria lysis occurs due to lower ruminal pH (Dong *et al.*, 2011). The presence of LPS may increase the permeability of the rumen wall and disrupt the rumen epithelial structure and barrier function (Khafipour *etal*., 2009a). Then, LPS can translocate across the rumen epithelia into the peripheral circulation, triggering systemic inflammation. Compared with the LCD, the HCD resulted in higher concentrations of plasma LPS, proinflammatory cytokines and APPs. Therefore, feeding HCD is not beneficial to the health of cattle.

It is noted that, in our present study, the ruminal pH values of the LCD and HCD (7.29 *v*. 6.77) were generally higher. The reason might be that the pH values were measured using the oral stomach tube sampling method at one time point, which did not average out the fluctuation of the rumen pH values.

In the present study, we also observed that feeding the HCD altered the ruminal bacterial composition. The HCD led to lower bacterial richness and diversity than the LCD. High richness and diversity in bacterial microbiota are considered beneficial, which can increase the stability of the rumen (Khafipour *et al.*, 2016). In this sense, the HCD may promote dysbiosis in the rumen. In the study of Khafipour *et al.* (2009b), when SARA was induced by increasing grain proportion in the diet of dairy cows, the percentage of ruminal Firmicutes increased and that of Bacteroidetes decreased. The abundance of Firmicutes increases the fermentation of carbohydrates and the lower number of Bacteroidetes may be explained by increased death and lysis due to lower pH values (Mao *etal*., 2013). At the genus level, *Prevotella* was the most abundant genus in the rumen fluid and decreased in the present study when cattle were fed the HCD, which is in concord with the result of Kim *et al.* who reported that *Prevotella* was predominant genus and significantly reduced in the rumen when cattle were fed HCD (Kim *etal*., 2018).

Acid treatment can change the physicochemical properties of starch (Humer and Zebeli, 2017). The cross-linking structure formed between starch and acid is able to resist enzyme digestion in the rumen (Van Hung *etal*., 2016). Our present study demonstrated that treatment of corn with 1% HA remarkably increased RS content. Resistant starch is fermented more slowly than NRS in the rumen, thus slows down the rapid accumulation of SCFAs in the rumen and increases ruminal pH (Shen *etal*., 2018). Our results showed that feeding the HCDT tended to decrease the total SCFAs and elevate the pH value compared with the HCD. Furthermore, compared with the HCD, the HCDT reduced the LPS in the rumen, resulting in decreased TNF-*α*, IL-6, SAA and Hp in the plasma. Moreover, in the monogastric animals, RS is mainly fermented as SCFAs in the hind gut (Iqbal *et al.*, 2009). In contrast, majority of RS is digested as glucose in the small intestine in ruminants (Iqbal *et al.*, 2009; Shen *et al.*, 2018). Therefore, high proportion of RS in ruminant diet supplies more efficient glucose that reaches the liver, as compared with gluconeogenesis from SCFAs in the liver (Deckardt *etal*., 2013). Reynolds *et al.* (2006) confirmed that an increase in the glucose absorption in small intestine was associated with an increase of milk yield in lactating dairy cows. Therefore, the presence of RS in the HCDT may help increase the glucose supply to promote the growth performance and, at the same time, lower the risk of ruminal acidosis.

The treatment of corn with HA also helped improve the ruminal bacterial communities in the HCDT group compared with the HCD group. In the HCDT group, the abundance of Bacteroidetes was higher than in the HCD group. We speculated that feeding the HCDT reduced the death of Bacteroidetes due to the presence of more RS which decreases the rapid accumulation of SCFAs in the rumen, resulting in the abundance of Bacteroidetes. Since Bacteroidetes are gram-negative bacteria, the reduced death of Bacteroidetes may explain the LPS content in rumen fluid and plasma decreased in the HCDT group compared with the HCD group. In the study of Shen *etal*. (2018), dairy goats fed a HCD based on corn treated with 0.5% citric acid had lower ruminal LPS concentration, which was due to less starch being digested in the rumen. As a result, less SCFAs were produced to preclude ruminal pH reduction, resulting in less deaths of bacteria including Bacteroidetes as observed in the present study. As mentioned above, the immune response can be activated when plasma LPS content increased, resulting in higher cytokine concentrations in the plasma. Cytokines are intercellular signaling polypeptides produced and participating in the inflammatory process and are the major stimulators for the production of APPs (Gabay and Kushner, 1999). Compared with the HCD group, TNF-α and IL-6 as well as Hp and SAA were significantly reduced in the HCDT group. Deckardt *etal*. (2013) reported similar results in dairy cows when they used lactic acid to treat barley. Furthermore, feeding the HCDT increased the alpha diversity indices and richness of the ruminal bacterial microbiota compared with feeding the HCD. The diversity and abundance of the ruminal bacterial community are beneficial to the health of the cattle (Khafipour *etal*., 2016).

Overall, on the basis of our results, we concluded that feeding the HCD promoted the pH reduction and the LPS release in the rumen. Furthermore, feeding the HCD increased inflammatory response and disturbed the ruminal bacterial stability. Treatment of corn with HA in combination with subsequent SB neutralization increased the RS content, modified rumen fermentation patterns and microbiota, and suppressed the negative effects of feeding HCD to beef steers.

## Software and data repository resources

The raw sequencing data were submitted to the Sequence Read Archive (SRA) of National Center for Biotechnology Information (NCBI) (http://www.ncbi.nlm.nih.gov/sra) under the accession number of PRJNA545031.

## Supplementary material

For supplementary material accompanying this paper visit https://doi.org/10.1017/S1751731120000786. click here to view supplementary material
