# Effects of the acid–base treatment of corn on rumen fermentation and microbiota, inflammatory response and growth performance in beef cattle fed high-concentrate diet – CORRIGENDUM

**DOI:** 10.1017/S175173112000172X

**Published:** 2020-11

**Authors:** J. Liu, K. Tian, Y. Sun, Y. Wu, J. Chen, R. Zhang, T. He, G. Dong

**Keywords:** hydrochloric acid, sodium bicarbonate, ruminal bacteria, lipopolysaccharide, steer, corrigendum

The original publication contained an error in Table 3. The corrected version of Table 3 is shown here;

**Table 3:** The plasma acute phase protein (APP) and proinflammatory cytokine levels in beef cattle fed different diets

Item	Diets^1^			SEM	*P*-values
	LCD	HCD	HCDT		
APP					
LBP (μg/ml)	298^a^	324^b^	309^ab^	5.1	0.013
SAA (μg/ml)	13.7^A^	15.3^B^	13.9^A^	0.24	0.006
CRP (mg/l)	9.4^a^	11.0^b^	9.8^ab^	0.26	0.035
Hp (μg/ml)	1809^a^	2119^b^	1798^a^	60.0	0.034
Proinflammatory cytokine					
TNF-α (pg/ml)	1209^a^	1430^Bb^	1233^Aa^	34.8	0.008
IL-1β (pg/ml)	134^a^	159^b^	157^b^	4.3	0.026
IL-6 (pg/ml)	298^a^	322^b^	298^a^	4.5	0.038
IL-8 (pg/ml)	291^a^	325^b^	306^ab^	5.9	0.046

LBP = lipopolysaccharide-binding protein; SAA = serum amyloid A; CRP = C-reactive protein; Hp = haptoglobin; TNF-α = tumor necrosis factor α; IL-1β = interleukin-1β; IL-6 = interleukin-6; IL-8 = interleukin-8.

1 LCD, low-concentrate diet based on corn steeped in tap water for 48 h; HCD, high-concentrate diet based on corn steeped in tap water for 48 h; HCDT, high-concentrate diet based on corn steeped in 1% (wt/wt) hydrochloric acid for 48 h in combination with subsequent sodium bicarbonate neutralization.

^A,B^Means of the same row not sharing an uppercase letter differ (*P* < 0.01).

^a,b^Means of the same row not sharing a lowercase letter differ (*P* < 0.05).

The authors apologise for the error.
