# Supplementary material for: Effects of the acid–base treatment of corn on rumen fermentation and microbiota, inflammatory response and growth performance in beef cattle fed high-concentrate diet
Source: Animal. 2020 Apr 16;14(9):1876–84. doi: 10.1017/S1751731120000786 (PMC7435151; doi:10.1017/S1751731120000786)
Supplement: Supplementary file 1 [file S1751731120000786sup001.docx]

**Effects of the acid-base treatment of corn on rumen fermentation and microbiota, inflammatory response** **and growth performance in beef cattle fed high-concentrate diet**

Junhui Liu, Ke Tian, Yawang Sun, Yongjiang Wu, Juncai Chen, Ruiming Zhang, Tianle He and Guozhong Dong

Short title: Acid-base treatment of corn for steers

**Supplementary Figure S1** Unweighted UniFrace principal coordinate analysis (PCoA) of rumen fluid bacterial community when beef cattle were fed different diets. PC1, PCoA axis 1; PC2, PCoA axis 2; LCD, low-concentrate diet based on corn steeped in tap water for 48 h; HCD, high-concentrate diet based on corn steeped in tap water for 48 h; HCDT, high-concentrate diet based on corn steeped in 1% (wt/wt) hydrochloric acid for 48 h in combination with subsequent sodium bicarbonate neutralization.

**Supplementary Table S1** *Initial bacterial composition (%) in rumen fluid at the phylum level of each cattle (Rumen bacterial baseline)*

| Number^1^ | Bacteroidetes | Firmicutes | Proteobacteria | Tenericutes | Cyanobacteria | Spirochaetes | Fibrobacteres | Verrucomicrobia | TM7 | WPS-2 | Others |
| --- | --- | --- | --- | --- | --- | --- | --- | --- | --- | --- | --- |
| 1 | 58.32 | 33.29 | 5.67 | 0.67 | 1.07 | 0.30 | 0.01 | 0.24 | 0.04 | 0.00 | 0.39 |
| 2 | 56.15 | 35.30 | 6.26 | 0.54 | 0.95 | 0.24 | 0.02 | 0.10 | 0.11 | 0.00 | 0.33 |
| 3 | 54.03 | 37.66 | 4.35 | 0.50 | 0.02 | 0.36 | 0.70 | 1.90 | 0.02 | 0.01 | 0.44 |
| 4 | 53.02 | 40.17 | 4.44 | 0.74 | 0.43 | 0.21 | 0.04 | 0.00 | 0.03 | 0.02 | 0.90 |
| 5 | 51.82 | 41.69 | 5.06 | 0.12 | 0.02 | 0.11 | 0.49 | 0.11 | 0.00 | 0.19 | 0.39 |
| 6 | 61.15 | 31.02 | 6.65 | 0.58 | 0.06 | 0.39 | 0.01 | 0.04 | 0.00 | 0.05 | 0.05 |
| 7 | 60.90 | 31.38 | 6.61 | 0.55 | 0.06 | 0.04 | 0.06 | 0.04 | 0.03 | 0.00 | 0.33 |
| 8 | 58.03 | 34.75 | 4.06 | 0.47 | 0.03 | 1.24 | 0.37 | 0.45 | 0.05 | 0.17 | 0.37 |
| 9 | 57.71 | 35.88 | 3.69 | 0.85 | 0.00 | 0.47 | 0.45 | 0.34 | 0.03 | 0.28 | 0.29 |
| 10 | 57.09 | 36.66 | 4.56 | 0.57 | 0.07 | 0.27 | 0.09 | 0.02 | 0.16 | 0.01 | 0.49 |
| 11 | 62.63 | 31.19 | 3.58 | 0.55 | 0.38 | 0.69 | 0.28 | 0.21 | 0.09 | 0.00 | 0.39 |
| 12 | 55.72 | 36.86 | 4.61 | 0.98 | 0.16 | 0.32 | 0.50 | 0.36 | 0.00 | 0.02 | 0.46 |
| 13 | 50.60 | 43.68 | 4.47 | 0.11 | 0.01 | 0.36 | 0.32 | 0.08 | 0.06 | 0.06 | 0.24 |
| 14 | 50.97 | 39.31 | 7.92 | 0.80 | 0.20 | 0.10 | 0.09 | 0.09 | 0.03 | 0.00 | 0.48 |
| 15 | 57.82 | 32.90 | 7.45 | 0.13 | 0.09 | 0.20 | 0.37 | 0.41 | 0.00 | 0.08 | 0.55 |
| 16 | 61.16 | 33.02 | 4.45 | 0.58 | 0.11 | 0.19 | 0.01 | 0.08 | 0.00 | 0.07 | 0.33 |
| 17 | 62.47 | 30.10 | 5.32 | 0.90 | 0.28 | 0.10 | 0.00 | 0.02 | 0.02 | 0.00 | 0.79 |
| 18 | 58.77 | 34.81 | 5.17 | 0.18 | 0.05 | 0.03 | 0.00 | 0.03 | 0.66 | 0.01 | 0.29 |

^1^ Number of the experimental cattle in the present research.

**Supplementary Table S2** *Initial bacterial composition (%) in rumen fluid at the genus level of each cattle (Rumen bacterial baseline)*

| Number^1^ | Prevotella | Unclassified  Clostridiales | Succiniclasticum | Unclassified  RF16 | Unclassified  Bacteroidales | Unclassified  Ruminococcaceae | Unclassified  S24-7 | Unclassified  Succinivibrionaceae | Unclassified  Lachnospiraceae | Ruminococcus | Others |
| --- | --- | --- | --- | --- | --- | --- | --- | --- | --- | --- | --- |
| 1 | 39.69 | 6.04 | 5.85 | 6.46 | 4.62 | 5.21 | 3.11 | 6.06 | 3.27 | 3.61 | 16.05 |
| 2 | 40.00 | 6.42 | 5.37 | 7.85 | 6.35 | 3.24 | 3.24 | 6.04 | 2.42 | 3.78 | 15.29 |
| 3 | 40.14 | 7.63 | 6.45 | 6.10 | 6.12 | 3.56 | 3.27 | 7.66 | 2.48 | 2.01 | 14.58 |
| 4 | 39.88 | 6.89 | 7.93 | 6.80 | 7.76 | 2.88 | 2.44 | 5.58 | 2.88 | 2.09 | 14.88 |
| 5 | 40.69 | 6.24 | 7.50 | 8.04 | 7.06 | 5.57 | 4.19 | 4.18 | 2.89 | 2.75 | 10.88 |
| 6 | 35.08 | 4.12 | 6.63 | 7.58 | 5.70 | 5.02 | 5.60 | 7.88 | 4.54 | 3.81 | 14.05 |
| 7 | 38.37 | 7.60 | 7.21 | 8.58 | 7.49 | 5.64 | 3.25 | 5.22 | 2.74 | 2.07 | 11.83 |
| 8 | 34.19 | 6.50 | 8.75 | 7.58 | 4.37 | 6.76 | 5.75 | 5.31 | 4.06 | 3.34 | 13.38 |
| 9 | 36.15 | 8.90 | 5.33 | 6.22 | 5.15 | 5.79 | 4.43 | 5.33 | 4.89 | 4.00 | 13.80 |
| 10 | 36.88 | 8.49 | 5.75 | 6.88 | 7.49 | 3.49 | 3.50 | 7.32 | 2.01 | 2.81 | 15.39 |
| 11 | 41.18 | 6.03 | 5.40 | 6.78 | 5.02 | 2.77 | 3.79 | 4.69 | 4.76 | 4.04 | 15.55 |
| 12 | 38.57 | 7.92 | 8.11 | 5.22 | 6.77 | 5.23 | 4.03 | 4.13 | 4.04 | 3.11 | 12.87 |
| 13 | 38.38 | 5.42 | 5.41 | 7.81 | 6.64 | 3.59 | 3.59 | 6.91 | 2.48 | 3.59 | 16.17 |
| 14 | 40.76 | 7.05 | 6.45 | 6.77 | 4.22 | 6.84 | 3.42 | 6.53 | 2.64 | 2.52 | 12.79 |
| 15 | 40.32 | 6.37 | 5.17 | 5.94 | 6.26 | 5.87 | 5.53 | 5.06 | 4.15 | 2.46 | 12.87 |
| 16 | 34.05 | 6.27 | 6.09 | 5.23 | 7.61 | 4.32 | 4.92 | 6.69 | 5.25 | 2.42 | 17.14 |
| 17 | 43.17 | 7.06 | 5.44 | 5.52 | 4.49 | 3.04 | 4.36 | 5.42 | 4.38 | 3.59 | 13.53 |
| 18 | 36.31 | 5.70 | 5.87 | 6.37 | 5.60 | 5.97 | 3.46 | 5.49 | 4.60 | 2.96 | 17.68 |

^1^ Number of the experimental cattle in the present research.

**Supplementary Table S3** *Phylum level composition (%) of rumen fluid bacterial community of beef cattle fed different diets*

| Phylum | Diet | | | SEM | *P* value |
| --- | --- | --- | --- | --- | --- |
|  | LCD^1^ | HCD | HCDT |  |  |
| Firmicutes | 43.57^A^ | 56.50^B^ | 47.11^A^ | 1.724 | 0.001 |
| Bacteroidetes | 50.37^Aa^ | 35.02^B^ | 45.23^Cb^ | 1.846 | <0.001 |
| Spirochaetes | 1.40 | 1.30 | 2.05 | 0.261 | 0.500 |
| Actinobacteria | 0.30^Aa^ | 2.80^B^ | 0.62^b^ | 0.426 | 0.017 |
| Proteobacteria | 0.80 | 1.06 | 2.43 | 0.356 | 0.153 |
| Tenericutes | 1.22 | 0.82 | 0.49 | 0.205 | 0.382 |
| Fibrobacteres | 0.95 | 0.64 | 0.42 | 0.135 | 0.311 |
| Verrucomicrobia | 0.52 | 0.41 | 0.95 | 0.142 | 0.304 |
| TM7 | 0.52 | 0.53 | 0.08 | 0.122 | 0.271 |
| WPS-2 | 0.09 | 0.09 | 0.22 | 0.048 | 0.534 |
| Other | 0.09 | 0.60 | 0.26 | 0.211 | 0.620 |
| Cyanobacteria | 0.05^a^ | 0.12^Bb^ | 0.01^A^ | 0.015 | 0.006 |
| Synergistetes | 0.05 | 0.02 | 0.04 | 0.008 | 0.262 |
| Lentisphaerae | 0.03 | 0.04 | 0.03 | 0.015 | 0.984 |
| SR1 | 0.02 | 0.03 | 0.04 | 0.009 | 0.843 |
| Fusobacteria | 0.01 | 0.04 | 0.01 | 0.007 | 0.110 |
| Elusimicrobia | 0.01 | 0.01 | 0.02 | 0.005 | 0.503 |

^1^ LCD, low-concentrate diet based on corn steeped in tap water for 48 h; HCD, high-concentrate diet based on corn steeped in tap water for 48 h; HCDT, high-concentrate diet based on corn steeped in 1% (wt/wt) hydrochloric acid for 48 h in combination with subsequent sodium bicarbonate neutralization.

^A-C^ Means of the same row not sharing an uppercase letter differ (*P* < 0.01).

^a-b^ Means of the same row not sharing a lowercase letter differ (*P* < 0.05).

**Supplementary Table S4** *Genus level composition (%) of rumen fluid bacterial community of beef cattle fed different diets*

| Genus | Diet | | | SEM | *P* value |
| --- | --- | --- | --- | --- | --- |
|  | LCD^1^ | HCD | HCDT |  |  |
| *Prevotella* | 31.17^A^ | 16.88^B^ | 19.46^B^ | 1.679 | <0.001 |
| Unclassified Bacteroidales | 9.70^A^ | 15.21^B^ | 16.31^B^ | 0.831 | <0.001 |
| *Succiniclasticum* | 8.50^A^ | 15.25^B^ | 14.26^B^ | 0.890 | <0.001 |
| Unclassified Clostridiales | 10.09 | 9.50 | 8.91 | 0.357 | 0.445 |
| Unclassified Ruminococcaceae | 10.17^A^ | 5.10^B^ | 9.36^A^ | 0.652 | <0.001 |
| *Ruminococcus* | 7.00 | 5.98 | 6.30 | 0.498 | 0.706 |
| *Treponema* | 0.62 | 0.66 | 0.87 | 0.057 | 0.179 |
| Unclassified S24-7 | 3.90^AB^ | 5.64^A^ | 2.76^B^ | 0.460 | 0.026 |
| Unclassified Lachnospiraceae | 1.99^ab^ | 3.09^a^ | 1.69^b^ | 0.253 | 0.046 |
| *Butyrivibrio* | 0.73^Aa^ | 2.84^B^ | 2.36^b^ | 0.313 | 0.004 |
| Unclassified RF16 | 2.92 | 2.09 | 2.26 | 0.330 | 0.575 |
| Unclassified Coriobacteriaceae | 0.04 | 2.00 | 0.16 | 0.495 | 0.187 |
| *Sharpea* | 0.05 | 2.29 | 2.16 | 0.734 | 0.386 |
| Unclassified BS11 | 1.30 | 0.56 | 0.57 | 0.186 | 0.175 |
| Unclassified Succinivibrionaceae | 0.29 | 1.76 | 1.48 | 0.628 | 0.615 |
| *Clostridium* | 0.11 | 1.69 | 0.33 | 0.548 | 0.457 |
| Unclassified RF39 | 1.02 | 0.75 | 0.38 | 0.212 | 0.516 |
| CF231 | 1.38^Aa^ | 0.13^B^ | 0.57^b^ | 0.174 | 0.002 |
| *Bulleidia* | 0.06 | 0.49 | 0.36 | 0.095 | 0.148 |
| *Fibrobacter* | 0.94 | 0.50 | 0.42 | 0.116 | 0.138 |
| *Moryella* | 0.62 | 0.64 | 0.72 | 0.068 | 0.851 |
| *Asteroleplasma* | 0.95 | 0.07 | 0.25 | 0.224 | 0.234 |
| Unclassified F16 | 0.52 | 0.53 | 0.08 | 0.122 | 0.271 |
| Unclassified Christensenellaceae | 0.52^a^ | 0.17^b^ | 0.39^ab^ | 0.070 | 0.010 |
| Unclassified Bifidobacteriaceae | 0.01 | 0.82 | 0.27 | 0.199 | 0.232 |
| *Coprococcus* | 0.26 | 0.32 | 0.37 | 0.077 | 0.842 |
| Other | 0.09 | 0.60 | 0.26 | 0.211 | 0.621 |
| Unclassified Veillonellaceae | 0.32 | 0.25 | 0.36 | 0.072 | 0.852 |
| *Bifidobacterium* | 0.22 | 0.45 | 0.22 | 0.073 | 0.334 |
| Unclassified WCHB1-25 | 0.14 | 0.24 | 0.52 | 0.110 | 0.383 |
| Unclassified [Mogibacteriaceae] | 0.47^a^ | 0.14^b^ | 0.24^ab^ | 0.056 | 0.028 |
| *Selenomonas* | 0.28 | 0.34 | 0.21 | 0.059 | 0.705 |
| *Megasphaera* | 0.05^a^ | 0.12^a^ | 0.46^b^ | 0.072 | 0.048 |
| *Sphaerochaeta* | 0.12 | 0.09 | 0.65 | 0.133 | 0.177 |
| Unclassified RFP12 | 0.37 | 0.11 | 0.20 | 0.066 | 0.246 |
| Unclassified [Paraprevotellaceae] | 0.40^a^ | 0.13^b^ | 0.20^ab^ | 0.047 | 0.034 |
| *RFN20* | 0.19 | 0.16 | 0.46 | 0.064 | 0.123 |
| *Oscillospira* | 0.25 | 0.18 | 0.25 | 0.035 | 0.662 |
| Unclassified GMD14H09 | 0.16 | 0.08 | 0.45 | 0.100 | 0.335 |
| *Anaerovibrio* | 0.15 | 0.19 | 0.23 | 0.042 | 0.739 |
| *Desulfovibrio* | 0.21 | 0.13 | 0.15 | 0.022 | 0.385 |
| *YRC22* | 0.11 | 0.11 | 0.21 | 0.038 | 0.538 |
| *BF311* | 0.20 | 0.14 | 0.12 | 0.040 | 0.736 |
| Unclassified WPS-2 | 0.09 | 0.08 | 0.21 | 0.048 | 0.482 |
| *Blautia* | 0.01 | 0.25 | 0.17 | 0.055 | 0.181 |
| *Anaeroplasma* | 0.17 | 0.05 | 0.07 | 0.030 | 0.194 |
| *Anaerostipes* | 0.13 | 0.04 | 0.09 | 0.025 | 0.344 |
| *Shuttleworthia* | 0.08 | 0.02 | 0.18 | 0.051 | 0.471 |
| *Pseudobutyrivibrio* | 0.06 | 0.09 | 0.09 | 0.019 | 0.821 |
| [Prevotella] | 0.07 | 0.07 | 0.03 | 0.017 | 0.517 |
| *Mogibacterium* | 0.09 | 0.03 | 0.04 | 0.016 | 0.372 |
| Unclassified Prevotellaceae | 0.15 | < 0.01 | < 0.01 | 0.039 | 0.211 |
| Unclassified Oxalobacteraceae | 0.05 | 0.06 | 0.06 | 0.017 | 0.976 |
| *Ruminobacter* | 0.01 | 0.05 | 0.10 | 0.020 | 0.211 |
| Unclassified Streptophyta | 0.05^a^ | 0.09^Ab^ | < 0.01^Bc^ | 0.012 | 0.002 |
| *Succinivibrio* | < 0.01 | 0.01 | 0.13 | 0.029 | 0.161 |
| *p-75-a5* | 0.04 | 0.05 | 0.02 | 0.007 | 0.143 |
| Unclassified Clostridiaceae | 0.05 | 0.02 | 0.03 | 0.007 | 0.111 |
| Unclassified Victivallaceae | 0.03 | 0.04 | 0.03 | 0.015 | 0.979 |
| *Pyramidobacter* | 0.05 | 0.01 | 0.04 | 0.008 | 0.242 |
| Unclassified SR1 | 0.02 | 0.03 | 0.04 | 0.009 | 0.857 |
| Unclassified Rickettsiales | 0.02 | 0.04 | 0.02 | 0.011 | 0.731 |
| *Dialister* | < 0.01 | 0.07 | 0.01 | 0.024 | 0.469 |
| *Acidaminococcus* | < 0.01 | 0.03 | 0.06 | 0.014 | 0.304 |
| *L7A E11* | 0.04 | 0.02 | 0.01 | 0.007 | 0.396 |
| *Mitsuokella* | < 0.01 | 0.04 | 0.03 | 0.010 | 0.194 |
| Unclassified RF32 | 0.01 | 0.02 | 0.04 | 0.013 | 0.618 |
| Unclassified YS2 | 0.01 | 0.04 | 0.01 | 0.008 | 0.090 |
| *Pseudoramibacter Eubacterium* | < 0.01^a^ | 0.01^ab^ | 0.04^b^ | 0.007 | 0.042 |
| Unclassified Elusimicrobiaceae | 0.01 | 0.01 | 0.03 | 0.006 | 0.388 |
| *Lactobacillus* | 0.04^a^ | < 0.01^b^ | < 0.01^b^ | 0.008 | 0.043 |
| *Desulfobulbus* | 0.01 | 0.02 | 0.02 | 0.005 | 0.774 |
| Unclassified Burkholderiales | 0.02 | 0.01 | 0.01 | 0.004 | 0.423 |
| *Roseburia* | < 0.01 | 0.03 | 0.01 | 0.007 | 0.188 |
| Unclassified Sphaerochaetaceae | < 0.01 | 0.02 | 0.01 | 0.007 | 0.369 |
| Unclassified Mycoplasmataceae | 0.01 | 0.02 | < 0.01 | 0.004 | 0.311 |
| *Fusobacterium* | 0.01^a^ | 0.02^b^ | < 0.01^a^ | 0.004 | 0.040 |
| Unclassified WCHB1-41 | 0.01 | < 0.01 | 0.02 | 0.005 | 0.153 |
| *Dorea* | 0.01 | < 0.01 | 0.02 | 0.005 | 0.429 |
| Unclassified Desulfovibrionaceae | 0.01 | 0.02 | < 0.01 | 0.004 | 0.267 |
| *Leuconostoc* | < 0.01^A^ | 0.02^Bb^ | < 0.01^a^ | 0.003 | 0.020 |
| *Suttonella* | < 0.01 | 0.02 | < 0.01 | 0.006 | 0.126 |
| Unclassified p-2534-18B5 | 0.01 | < 0.01 | 0.02 | 0.005 | 0.385 |
| Unclassified Acholeplasmatales | 0.02 | < 0.01 | < 0.01 | 0.007 | 0.297 |
| Unclassified Actinomycetaceae | 0.01 | 0.01 | < 0.01 | 0.003 | 0.547 |
| Unclassified Lactobacillales | < 0.01 | 0.01 | 0.01 | 0.003 | 0.467 |
| Unclassified Leptotrichiaceae | 0.01 | 0.01 | < 0.01 | 0.005 | 0.630 |
| *Corynebacterium* | 0.01 | < 0.01 | < 0.01 | 0.003 | 0.335 |
| Unclassified Spirochaetaceae | < 0.01 | 0.01 | 0.01 | 0.003 | 0.391 |
| *Atopobium* | 0.01 | 0.01 | < 0.01 | 0.002 | 0.601 |
| *PSB-M-3* | < 0.01 | 0.01 | < 0.01 | 0.002 | 0.280 |
| *Cetobacterium* | < 0.01 | < 0.01 | 0.01 | 0.003 | 0.515 |
| [Ruminococcus] | < 0.01 | 0.01 | < 0.01 | 0.002 | 0.516 |
| *Streptococcus* | 0.01 | < 0.01 | < 0.01 | 0.003 | 0.734 |
| [Eubacterium] | < 0.01 | 0.01 | < 0.01 | 0.003 | 0.150 |
| *TG5* | < 0.01 | < 0.01 | < 0.01 | 0.001 | 0.815 |
| Unclassified 0319-6G20 | 0.01 | < 0.01 | < 0.01 | 0.003 | 0.180 |
| Unclassified Chitinophagaceae | 0.01 | < 0.01 | < 0.01 | 0.003 | 0.503 |
| *Lachnobacterium* | < 0.01 | 0.01 | < 0.01 | 0.003 | 0.427 |
| *Adlercreutzia* | < 0.01 | < 0.01 | < 0.01 | 0.002 | 0.579 |
| *Cupriavidus* | < 0.01 | < 0.01 | < 0.01 | 0.001 | 0.615 |
| *Campylobacter* | < 0.01 | 0.01 | < 0.01 | 0.001 | 0.181 |
| Unclassified Comamonadaceae | < 0.01 | 0.01 | < 0.01 | 0.001 | 0.172 |
| *Paludibacter* | < 0.01 | < 0.01 | < 0.01 | 0.001 | 0.321 |
| Unclassified R4-45B | < 0.01 | < 0.01 | < 0.01 | 0.001 | 0.427 |
| Unclassified Enterobacteriaceae | < 0.01 | < 0.01 | < 0.01 | 0.001 | 0.141 |
| Unclassified Acetobacteraceae | < 0.01 | < 0.01 | < 0.01 | 0.001 | 0.588 |
| *Microbacterium* | < 0.01 | < 0.01 | < 0.01 | 0.001 | 0.321 |
| Unclassified Peptostreptococcaceae | < 0.01 | < 0.01 | < 0.01 | 0.001 | 0.427 |
| *Syntrophococcus* | < 0.01 | < 0.01 | < 0.01 | 0.001 | 0.427 |
| Unclassified HA64 | < 0.01 | < 0.01 | < 0.01 | 0.001 | 0.427 |
| *Akkermansia* | < 0.01 | < 0.01 | < 0.01 | 0.001 | 0.321 |

^1^ LCD, low-concentrate diet based on corn steeped in tap water for 48 h; HCD, high-concentrate diet based on corn steeped in tap water for 48 h; HCDT, high-concentrate diet based on corn steeped in 1% (wt/wt) hydrochloric acid for 48 h in combination and with subsequent sodium bicarbonate neutralization.

^A-B^ Means of the same row not sharing an uppercase letter differ (*P* < 0.01).

^a-c^ Means of the same row not sharing a lowercase letter differ (*P* < 0.05).
